# Pain in Alzheimer’s Disease: Disrupted Multilevel Integration of Nociception, Affective Processing and Clinical Expression Across Clinical and Preclinical Evidence

**DOI:** 10.3390/life16050860

**Published:** 2026-05-21

**Authors:** Gabriela-Dumitrita Stanciu, Ivona Costachescu, Raluca-Maria Gogu, Bogdan-Ionel Tamba

**Affiliations:** 1 Advanced Research and Development Center for Experimental Medicine “Prof. Ostin C. Mungiu”-CEMEX, Grigore T. Popa University of Medicine and Pharmacy Iasi, 700454 Iasi, Romania; 2 Department of Pharmacology, Clinical Pharmacology and Algesiology, Grigore T. Popa University of Medicine and Pharmacy Iasi, 700115 Iasi, Romania

**Keywords:** Alzheimer’s disease, multilevel integration, clinical–preclinical translation, core mechanism, pain assessment, neurodegeneration

## Abstract

Pain is a multidimensional experience arising from the integration of nociceptive signals with affective, cognitive and behavioral processes. In Alzheimer’s disease (AD), pain assessment remains challenging, as reduced self-reported pain is frequently observed despite exposure to potentially painful conditions, suggesting altered processing rather than its absence. Emerging evidence indicates that pain in AD is characterized by a disruption of coordination among sensory detection, affective experience and clinical expression. Within this framework, nociceptive input may remain partially preserved, while its integration into emotionally meaningful and behaviorally coherent responses is compromised. Clinical studies report reduced self-report alongside observable indicators of discomfort, including agitation, withdrawal and affective disturbances. In parallel, preclinical models demonstrate preserved reflexive responses but altered affective-motivational processing. These alterations are associated with neuroinflammatory processes, synaptic dysfunction, large-scale network disconnection and changes in neuromodulatory systems involved in affective pain regulation, ultimately disrupting the integration of nociceptive signals within limbic and cortical networks. Taken together, this review integrates clinical and preclinical evidence to characterize pain in AD as a disruption of multilevel integration linking nociception, affective processing and clinical expression, with important implications for pain assessment strategies that extend beyond self-report to incorporate behavioral and translational approaches.

## 1. Introduction

Pain, as defined by the International Association for the Study of Pain (IASP), is “an unpleasant sensory and emotional experience associated with actual or potential tissue damage, or described in terms of such damage” [1]. This definition reflects the inherently multidimensional nature of pain, arising from the integration of nociceptive input with affective, cognitive and related processes, rather than representing a purely sensory phenomenon. At the systems level, pain is a distributed neurobiological process involving ascending nociceptive pathways and large-scale cortical–subcortical networks [2,3]. Regions including the insula, anterior cingulate cortex, amygdala and prefrontal cortex contribute to the integration of sensory-discriminative, affective-motivational and evaluative components of pain [4,5,6,7]. Within this framework, pain emerges as a constructed experience resulting from multilevel neural integration, rather than a direct representation of nociceptive activity [2,3].

Pain represents a highly prevalent clinical burden in older adults [8,9,10], with chronic pain affecting approximately 25–50% of community-dwelling individuals and up to 80% of institutionalized populations [11,12,13]. In neurocognitive disorders, pain assessment is further complicated by impaired communication and reduced reliability of self-report, contributing to frequent under-recognition. Persistent, unaddressed pain may exacerbate cognitive dysfunction and manifest as behavioral disturbances, including agitation, restlessness and social withdrawal [14,15]. These limitations underscore the need for validated observational instruments, such as the Pain Assessment in Advanced Dementia (PAIN-AD) and the Pain Assessment Checklist for Seniors with Limited Ability to Communicate (PACSLAC), to improve clinical detection [16].

In Alzheimer’s disease (AD), neurodegeneration extends beyond memory systems, involving networks critical for salience processing, emotional regulation, and behavioral control [17,18]. Consequently, pain assessment becomes increasingly indirect and less reliable in advanced stages of the disease [17,19,20]. Clinical studies consistently report reduced self-reported pain intensity in AD compared to cognitively intact older adults, despite exposure to comparable nociceptive conditions [21,22]. While traditionally interpreted as reduced pain sensitivity, this observation is increasingly questioned in light of behavioral and translational evidence [23,24]. Specifically, observational studies using behavioral pain-assessment instruments, including the PAIN-AD, PACSLAC, the Mobilization–Observation–Behaviour–Intensity–Dementia (MOBID) Pain and the Abbey Pain (APS) scales, demonstrate that individuals with AD may continue to exhibit pain-related facial expressions, vocalizations, protective body movements and changes in interpersonal behavior, despite reduced verbal pain reporting [25,26]. In parallel, preclinical animal studies support a dissociation between nociceptive processing and affective-motivational responses, indicating a multilevel disruption of pain integration [27,28]. Experimental rodent models of acute inflammatory pain and repetitive pain exposure have demonstrated alterations in anxiety-related and attentional responses, despite relatively preserved nociceptive reactivity, suggesting that affective and behavioral dimensions of pain may be differentially modulated from primary sensory processing [27,28]. Similarly, transgenic AD models, including APP/PS1 mice, have shown altered pain-related and cognitive–affective responses following inflammatory or chronic pain models, further supporting the possibility that AD-related neurodegeneration disrupts higher-order integration of pain processing [29].

These converging findings raise a key unresolved question regarding the mechanisms underlying altered pain expression in AD, particularly as to whether they reflect altered sensory processing, impaired affective integration or dysfunction within higher-order networks responsible for generating coherent clinical responses. While previous reviews have examined sensory–affective aspects of pain processing in AD and related neurocognitive disorders [30,31,32], several questions remain regarding how alterations across nociceptive, affective and behavioral domains relate to the broader neurodegenerative changes characteristic of AD. Building on these earlier frameworks, the present work integrates findings from both clinical and preclinical studies to examine pain alterations within a multilevel perspective encompassing nociceptive processing, affective-motivational mechanisms and clinical pain expression. In doing so, the review aims to highlight how dysfunction across distributed cortical–subcortical networks may contribute to discrepancies between nociceptive signaling, subjective report and observable pain-related behaviors in AD.

## 2. Mechanistic Substrates of Disrupted Nociceptive–Affective Integration in Alzheimer’s Disease

**Figure 1:**
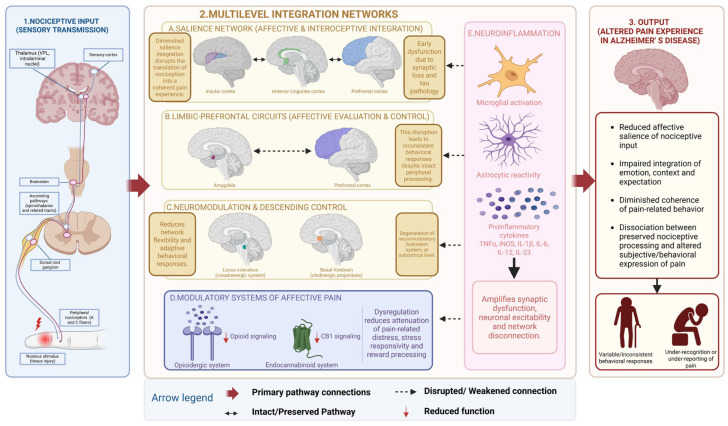
Multilevel neural mechanisms underlying altered pain processing in Alzheimer’s disease. (1) Nociceptive input (sensory transmission): peripheral nociceptive stimuli are transmitted from nociceptors through ascending spinothalamic pathways to thalamic and cortical regions involved in sensory-discriminative pain processing. Preserved primary nociceptive transmission may coexist with altered higher-order pain integration in AD. (2) Multilevel integration networks: pain-related information is integrated across distributed cortical–subcortical systems involved in salience attribution, emotional evaluation, behavioral regulation and pain modulation. (**A**) The salience network, including the insular cortex, anterior cingulate cortex and prefrontal cortex, contributes to interoceptive awareness and affective integration of pain. (**B**) Limbic–prefrontal and reward processing circuits mediate emotional appraisal, contextual interpretation and behavioral control of pain responses. (**C**) Neuromodulatory and descending control systems, including locus coeruleus and basal forebrain projections, regulate nociceptive gain and adaptive behavioral responses. (**D**) Modulatory systems of affective pain, including opioidergic and endocannabinoid signaling pathways, contribute to stress regulation, emotional responsiveness and reward-related modulation of pain. (**E**) Neuroinflammatory mechanisms, including microglial activation, astrocytic reactivity and proinflammatory cytokine release, may further amplify synaptic dysfunction and network disconnection. (3) Output—altered pain experience in Alzheimer’s disease. The figure was created in BioRender. Abbreviations: TNF-α, tumor necrosis factor-α; iNOS, inducible Nitric Oxide Synthase; IL-1β, Interleukin-1 beta; IL-6, Interleukin-6; IL-12, Interleukin-12; IL-23, Interleukin-23.

Pain modulation is mediated by distributed neural systems operating across multiple levels of organization, including cortical regions, limbic–subcortical circuits and brainstem pathways involved in descending control of nociceptive processing [33,34,35]. Within this framework, nociceptive signals conveyed via spinothalamic and related ascending pathways are relayed to thalamocortical structures, which distribute sensory input to cortical regions [33,34,35]. However, the emergence of pain as a conscious and affectively meaningful experience is thought to depend on higher-order integration within the insular cortex, the anterior cingulate cortex, amygdala and prefrontal cortices. These regions form functionally interconnected networks that support salience attribution, affective valuation, interoceptive representation, and context-dependent appraisal of bodily states [4,5,6,7,34,35,36,37,38] (Figure 1). Notably, several of these alterations are thought to emerge during prodromal and early symptomatic stages of AD, when synaptic dysfunction and network-level disconnection may precede extensive neuronal loss [33,34]. Functional imaging and neuropathological studies suggest that salience-network regions, including the anterior cingulate and insular cortices, exhibit early vulnerability to tau-related pathology and altered functional connectivity [39,40,41,42,43]. As the disease progresses, pathology may extend to broader limbic and prefrontal networks, contributing to increasingly pronounced disturbances in affective integration, behavioral regulation and contextual evaluation of nociceptive stimuli [42,43]. Dysfunction within these networks is thought to compromise the attribution of affective relevance to incoming stimuli, including nociceptive mechanisms, thereby attenuating their motivational and emotional salience. As a consequence, nociceptive signals may be processed at a sensory level but fail to consistently engage affective-motivational systems required for the generation of a coherent subjective pain experience [42,43,44,45] (Figure 1).

Beyond salience-network dysfunction, progressive disconnection within limbic–prefrontal circuits further impair integrative processing. The amygdala–prefrontal axis is critical for linking affective evaluation with executive control, behavioral selection, and contextual modulation [46,47]. Degeneration of this circuitry in Alzheimer’s disease reduces top-down regulatory influences on nociceptive processing, limiting the integration of memory, expectation and contextual cues into pain evaluation. This disruption may contribute to variability and reduced coherence of behavioral responses to nociceptive stimuli, even when peripheral response remains intact [48,49] (Figure 1).

At the subcortical level, degeneration of neuromodulatory brainstem systems may further contribute to impaired network-level integration. The locus coeruleus–noradrenergic system and basal forebrain cholinergic projections regulate cortical excitability, attentional allocation, and descending inhibitory control of nociceptive transmission [50]. Their early vulnerability in AD has been proposed to contribute to reduced neuromodulatory signaling, thereby impairing gain-control mechanisms that normally regulate nociceptive processing in a context-dependent manner. This may result in reduced flexibility of pain-related network activity and a diminished capacity to adapt behavioral responses to internal states and environmental demands [51] (Figure 1). Complementing these brainstem systems, opioidergic and endocannabinoid pathways further modulate affective-motivational aspects of pain, including aversive salience, stress responsivity, and reward-related processing. Opioidergic signaling within limbic and prefrontal regions contributes to the attenuation of pain-related distress, while endocannabinoid signaling regulates synaptic transmission and emotional responsivity across cortico-limbic circuits [52,53]. In AD, dysregulation of these systems may arise through synaptic loss, degeneration of limbic–prefrontal networks, and altered neuromodulatory tone, potentially accompanied by changes in receptor expression and functional connectivity. These alterations may impair affective valuation and limit the integration of nociceptive input into coherent emotional and behavioral responses, thereby contributing to the dissociation between preserved nociceptive processing and altered clinical pain expression [33,50,51,52] (Figure 1). Importantly, these neuromodulatory and neurochemical systems converge on shared cortico-limbic networks, suggesting that their dysregulation may act synergistically, rather than independently, in shaping pain-related network dysfunction in AD.

Neuroinflammatory processes are also likely to interact with these network-level alterations. In AD, large-scale neural networks with high integrative and metabolic demands—particularly salience, limbic and prefrontal systems—appear especially vulnerable to early synaptic dysfunction, altered connectivity and progressive neurodegeneration [47,48,49]. Inflammatory signaling and structural degeneration appear to evolve in a partially overlapping manner across disease progression. Experimental and clinical evidence suggests that early microglial activation and cytokine dysregulation may emerge during prodromal stages, potentially preceding extensive neuronal loss and contributing to synaptic dysfunction and altered network connectivity [29,43,47,48,49]. As the disease progresses, structural degeneration and inflammatory signaling may increasingly reinforce one another through feed-forward mechanisms that promote network instability and impaired functional integration [47]. Microglial activation, astrocytic reactivity and cytokine-mediated signaling further modulate synaptic efficacy and neuronal excitability within pain-related circuits [54]. Rather than representing independent mechanisms, neuroinflammation may amplify pre-existing network vulnerabilities, establishing a self-reinforcing loop between structural and inflammatory processes that accelerates network breakdown [54,55] (Figure 1).

Collectively, these converging mechanisms support the hypothesis that altered pain expression in Alzheimer’s disease does not primarily arise from diminished nociceptive input alone, but from progressive disruption in the coordination of neural systems integrating sensory, affective and behavioral dimensions of pain. Within this framework, nociceptive signals may be detected and transmitted, yet their transformation into affectively meaningful and behaviorally coherent responses becomes increasingly unreliable. This may result in a partial decoupling between sensory encoding and clinical expression. Importantly, this integrative model generates testable predictions regarding pain processing in AD, including relatively preserved nociceptive reactivity, disproportionate impairment or variability in affective and behavioral responses, a progressive increase in this dissociation with disease severity, and reduced engagement of affective-motivational regions, despite preserved primary sensory-pathway activation. Together, these predictions provide a framework for interpreting heterogeneous and sometimes paradoxical findings across clinical and experimental studies of pain in Alzheimer’s disease.

## 3. Evidence for Dissociated Pain Processing in Alzheimer’s Disease: Clinical and Preclinical Perspectives

A growing body of evidence indicates that pain processing in Alzheimer’s disease cannot be adequately described as a uniform reduction in nociceptive sensitivity [34,56,57]. Instead, available data support a more nuanced reorganization of pain processing, characterized by a progressive dissociation between nociceptive encoding, affective integration and behavioral or verbal expression. This dissociation emerges consistently across clinical observations, experimental and preclinical models, suggesting that Alzheimer’s disease selectively disrupts higher-order integrative mechanisms while, relatively speaking, sparing primary sensory transmission [29,30,57].

### 3.1. Clinical Evidence: Dissociation Between Subjective Report and Behavioral Expression

In clinical populations, pain remains highly prevalent in AD, affecting approximately 45–75% of patients, with prevalence increasing in institutionalized settings where rates may reach up to 80%, depending on assessment methodology [58,59,60]. Despite this high burden, a consistent finding across studies is a reduction in self-reported pain intensity compared to cognitively intact older adults, even under comparable nociceptive conditions [61,62,63].

Importantly, this reduction in verbal report does not correspond to an absence of pain-related expression. On the contrary, observational studies consistently demonstrate the persistence of behavioral pain indicators, including facial motor responses, protective postures, vocalizations and alterations in social engagement. Instruments such as PAIN-AD and PACSLAC demonstrate moderate-to-good reliability, internal consistency and inter-rater agreement in patients with moderate-to-severe cognitive impairment, supporting their clinical utility when self-report becomes unreliable. Nevertheless, interpretation may be complicated by coexisting neuropsychiatric and medical comorbidities, including agitation, depression, delirium and motor impairment, which can overlap with behavioral indicators of pain [16,64,65]. Importantly, behavioral manifestations of pain are often amplified in contexts of advanced neuropsychiatric symptoms, where agitation, restlessness and withdrawal may partly reflect unrecognized pain states [66,67]. This divergence between subjective report and behavioral expression suggests that AD does not eliminate the experience of pain, but progressively alters its communicability and external readability. Pain, in this context, becomes increasingly decoupled from explicit awareness and linguistic representation, necessitating reliance on indirect behavioral markers. To provide a comprehensive overview, Table 1 summarizes representative clinical studies showing that pain thresholds are largely preserved in AD, with alterations in response dynamics and affective evaluation.

Across psychophysical paradigms, including thermal, mechanical and pressure-based stimulation, pain detection thresholds in Alzheimer’s disease are generally preserved, with only modest variability reported across studies [30,44,70,71]. This preservation suggests that early nociceptive transmission and basic sensory encoding remain relatively intact. However, higher-order aspects of pain processing reveal marked alterations. Individuals with AD frequently show delayed verbal responses to nociceptive stimuli and reduced consistency in subjective reporting [76]. Importantly, affective evaluations of pain unpleasantness are not uniformly diminished in AD and, in some studies, remain comparable to control populations, despite reduced self-reported pain intensity [77]. These findings support the possibility of a partial dissociation between sensory-discriminative and affective-motivational dimensions of pain processing in AD [78,79]. Neuroimaging studies provide complementary evidence, demonstrating relatively preserved activation in primary somatosensory cortices alongside altered engagement of regions involved in affective valuation and cognitive evaluation, including the anterior cingulate cortex, insula and prefrontal cortices [69,79]. Collectively, these findings support the view that Alzheimer’s disease does not uniformly impair pain processing but selectively disrupts the integration of nociceptive input into higher-order experiential and evaluative states.

### 3.2. Preclinical Evidence of Dissociated Pain Processing in Alzheimer and Chronic Pain Models

Preclinical evidence suggests that pain processing alterations associated with AD reflect a dissociation between relatively preserved sensory-discriminative components and disrupted affective-motivational and integrative cortical processing [58,59,60]. Importantly, the current literature comprises distinct categories of experimental models that provide complementary but fundamentally different types of evidence, with a relative predominance of studies focusing on classical chronic pain models compared to combined AD–pain models.

Classical chronic pain models in wild-type animals (e.g., neuropathic or inflammatory pain models) consistently demonstrate that persistent nociceptive input can induce impairments in learning, memory and affective behavior [79,80,81,82,83,84,85,86,87,88]. However, these effects reflect pain-induced alterations in brain function rather than Alzheimer’s disease-specific pathology. Accordingly, these studies are included as analogical and mechanistic evidence for pain–cognition interactions, providing insight into how sustained nociceptive signaling can impact cognitive and emotional processing in the absence of neurodegeneration [27,28,80,81,82,83,84,85,86,87,88].

Transgenic AD models, including APP/PS1, 3xTg-AD or 5 × FAD mice, provide direct evidence of how AD-related pathology influences nociceptive processing. Across these models, basic spinal- and brainstem-mediated nociceptive reflexes are generally preserved, indicating that primary sensory transmission remains largely intact [20,43]. In contrast, alterations emerge at higher-order levels of processing, involving cortical and limbic circuits responsible for integrating the emotional and behavioral significance of nociceptive stimuli [35,43]. In particular, dysfunction within regions such as the anterior cingulate cortex has been implicated in altered pain responsiveness, highlighting the role of synaptic- and network-level changes in pain integration during neurodegeneration [89].

Alzheimer’s disease models, combined with experimentally induced neuropathic or inflammatory pain, provide limited but mechanistically informative evidence for a bidirectional relationship between chronic nociceptive states and AD-related pathology. In these models, persistent pain has been shown to exacerbate cognitive deficits and is associated with increased neuroinflammation and aggravated amyloid-β and tau pathology, suggesting that chronic pain may contribute to the acceleration of disease-related neurobiological changes [89,90,91,92]. Representative studies supporting these categories are summarized in Table 2.

Taken together, these complementary lines of evidence support a unified framework in which pain processing in AD is characterized by a dissociation between preserved sensory nociception and altered affective-motivational and cortical integration. This framework further emphasizes that reduced or altered pain expression in AD likely arises from distributed network dysfunction affecting limbic–cortical circuits and synaptic processing within key integrative neural systems, rather than from changes in nociception alone.

## 4. Reconceptualizing Pain Assessment in Alzheimer’s Disease: Limitations and Future Integrative Frameworks

Pain assessment in Alzheimer’s disease is inherently constrained by its reliance on indirect proxies of a multidimensional experience that cannot be directly accessed [93,94,95]. While self-report remains the reference standard in cognitively intact individuals, its progressive loss in AD necessitates a shift toward observational and caregiver-based approaches [96,97,98,99]. However, growing evidence suggests that these methods capture pain only incompletely, as a distributed construct encompassing nociceptive processing, affective valuation and behavioral expression [97,98,99,100,101].

Observational scales such as PAIN-AD, PACSLAC, APS and DOLOPLUS-2 have substantially improved the clinical recognition of pain in individuals with moderate-to-severe dementia, particularly in contexts where self-report is no longer feasible [64,65,102,103]. These instruments operationalize pain through structured observation of facial expressions, vocalizations, body movements and changes in interpersonal behavior, thereby providing a pragmatic framework for bedside assessment [104]. However, their interpretative validity is constrained by limited specificity. Many of the behaviors captured—such as agitation, restlessness, vocal distress or withdrawal—are not unique to pain, but overlap extensively with broader neuropsychiatric manifestations of dementia, including anxiety, depression and BPSD [105,106,107,108]. This convergence introduces a critical ambiguity in clinical interpretation, as similar observable patterns may arise from distinct underlying processes. Consequently, elevated scores on observational scales do not necessarily reflect the intensity of nociceptive experience, but may instead index a combination of affective dysregulation, environmental stressors or unmet needs [108,109,110]. This challenge is further amplified in advanced stages of AD, where progressive cognitive and functional decline reduces both the diversity and clarity of behavioral expression, narrowing the observable repertoire through which pain and non-pain states alike can be inferred [104,105,106,107,108,109,110]. In addition to issues of construct specificity, methodological constraints further affect the reliability of these instruments. Variability across raters, differences in clinical training and contextual influences contribute to inconsistencies in scoring, while sensitivity to subtle or fluctuating pain states remains suboptimal [104]. Systematic evaluations have reported only moderate internal consistency and inter-rater reliability for commonly used tools, alongside heterogeneous performance across care settings. Taken together, these observations indicate that, although observational scales represent an essential component of current clinical practice, they provide a context-dependent approximation of pain in Alzheimer’s disease, rather than a direct measure [111,112]. Importantly, these limitations are not solely methodological, but also reflect a deeper conceptual challenge in how pain is understood and operationalized in this population.

Beyond this behavioral and methodological ambiguity, a more fundamental limitation lies in the implicit assumption that observable expression directly reflects subjective pain intensity. Converging evidence from clinical and experimental studies challenges this premise by demonstrating dissociations between nociceptive processing and behavioral output [113,114]. These findings have important implications for the validity of current assessment frameworks. Rather than capturing pain as a unified construct, existing tools are likely to reflect downstream manifestations of partially decoupled processes. Consequently, clinical interpretation relies heavily on contextual inference rather than direct measurement, increasing the risk of both under-recognition and misclassification of pain states.

Further complexity arises from practical and methodological constraints inherent to real-world assessment. Variability in caregiver training, environmental context, and patient state fluctuations introduces additional noise into observational ratings, reducing consistency across settings [115,116,117,118]. Moreover, the absence of a definitive biological or behavioral gold standard for pain in dementia limits the possibility of robust validation. As a result, many instruments are evaluated against other proxy-based or observational measures, giving rise to circular validation frameworks that constrain interpretability and comparability [119,120].

Recent advances in artificial intelligence and digital health technologies offer promising avenues for addressing the difficulty of accurately assessing pain in Alzheimer’s disease when nociceptive processing, affective integration and behavioral expression become partially dissociated [121,122,123]. Conventional observational scales often rely on overt behavioral outputs that may no longer reliably reflect underlying pain states in AD. In this context, machine learning approaches—particularly deep learning models applied to facial expression analysis, vocalization patterns and motor behavior—have demonstrated the capacity to detect pain-related states in individuals with dementia with increased sensitivity, higher temporal resolution and reduced observer bias [124,125,126,127,128]. Automated facial coding systems can detect subtle and transient micro-expressions that may not be consistently captured through conventional clinical assessment, while speech-based algorithms can extract paralinguistic signatures—including alterations in pitch variability, vocal intensity and prosodic modulation—associated with discomfort, affective distress and altered emotional processing [125,126,127,128,129].

In parallel, wearable sensors and multimodal digital biomarkers enable continuous monitoring of physiological correlates of pain, including heart rate variability, skin conductance, movement patterns and sleep–wake dynamics [130,131]. Importantly, the integration of behavioral, physiological and contextual data streams through data-driven models supports the characterization of pain as a dynamic and multidimensional process, rather than a static or isolated event [132,133]. From a conceptual perspective, these technologies align closely with the emerging view of pain in AD as a disruption of multilevel integration. By simultaneously capturing signals across sensory, affective and autonomic domains, AI-driven frameworks may help reconstruct latent pain states from distributed and partially decoupled outputs, thereby reducing some of the interpretative ambiguities inherent to conventional observational scales [121,122,123]. However, several challenges remain. Algorithm performance depends heavily on the quality and representativeness of training datasets, which are often limited in size and diversity, particularly in populations with advanced dementia. Variability in disease severity, comorbidities and cultural expression of pain further complicates model generalizability. In addition, issues related to data privacy, clinical interpretability and integration into routine-care workflows must be addressed before widespread implementation. Accordingly, while AI-based approaches represent a promising direction, their clinical utility will depend on rigorous validation, standardization and incorporation into clinically meaningful assessment frameworks [134,135].

Importantly, these technologies are not intended to replace existing clinical tools, but rather to augment them within a broader integrative framework. Rather than relying exclusively on single-domain measures or behavioral proxies, emerging approaches aim to approximate pain as a distributed construct arising from the interaction between nociceptive processing, affective modulation and observable behavior. Such a shift may improve sensitivity, reduce misclassification and ultimately support more accurate and responsive clinical decision-making in this vulnerable population.

## Figures and Tables

**Table 1:** Integrated clinical evidence of dissociated pain processing in Alzheimer’s disease.

Study	Design/Population	Pain Assessment	Key Findings	Clinical Significance
Prospective observational cohort [16]	Hospitalized older adults with major neurocognitive disorder (*n* = 292; mean age 87.8 ± 6.2 years)	PAIN-AD scale	Pain-related behaviors (PAIN-AD ≥ 2) in 62.4%; only 20.3% received analgesics; PAIN-AD associated with frailty, reduced quality of life and increased 3-month mortality (HR 2.39, 95% CI 1.20–4.74)	Pain is highly prevalent, under-treated, and independently predicts adverse functional outcomes and short-term mortality
Cross-sectional neuropsychological study with Lasso regression [22]	Memory clinic cohort (*n* = 179 individuals)	Self-reported pain (occurrence, intensity, severity, frequency)	Reduced memory and executive function associated with lower pain reporting; in pain reporters, memory deficits linked to lower pain ratings, while executive dysfunction showed inverse association (higher pain scores). Associations partially mediated by medial temporal lobe atrophy, white matter hyperintensities and depressive symptoms	Distinct cognitive domains differentially modulate pain reporting, suggesting that self-reported pain reflects an interaction between memory, executive control and affective processing, rather than pure nociceptive experience
Quasi-experimental video-based study in dementia care context [25]	*n* = 130: 65 nurses, 65 laypersons without healthcare training	PACSLAC-II and PAIN-AD observational scales applied to standardized pain/non-pain video stimuli	Both groups accurately differentiated pain from no-pain and graded intensity with no significant differences between raters; PACSLAC-II and PAIN-AD showed acceptable internal consistency (α = 0.69 and 0.61), acceptable split-half reliability (0.72 and 0.65), and high interrater reliability (ICC = 0.94–0.96)	Observational pain tools demonstrate robust reliability and validity across professional and lay users, supporting their utility for early and accessible pain detection in dementia care
Retrospective cross-sectional study [67]	Aged care residents with dementia (*n* = 479; mean age 81.9 ± 8.3 years)	PainChek^®^ combined with NPI	Pain identified in 65.6% of cases, with 48.4% moderate–severe; pain prevalence high across dementia subtypes (54.6–78.6%). Pain group showed higher neuropsychiatric symptom burden (+25.3% frequency, +33.6% severity) and caregiver distress (+31.4%); 3.8-fold increased likelihood of agitation/aggression	Pain is a major and underrecognized driver of BPSD in dementia, strongly associated with behavioral disturbance severity and caregiver burden, supporting its role as a modifiable clinical contributor to neuropsychiatric symptoms
Large-scale cross-sectional study [68]	Dementia referrals in residential aged care settings (immigrants *n* = 6340; non-immigrants *n* = 11,297)	PainChek^®^ and NPI	Similar pain prevalence across groups; immigrants showed slightly higher moderate–severe pain intensity (non-English-speaking immigrants: +0.5 PainChek^®^ points; Cohen’s d = 0.10). Pain intensity significantly associated with NPS severity across all subgroups	Pain expression and intensity in dementia are modulated by cultural background, while maintaining a consistent relationship with neuropsychiatric symptom burden, highlighting the need for culturally sensitive pain-assessment strategies
Experimental neuroimaging study using mechanical pressure-induced pain [69]	fMRI study in Alzheimer’s disease (*n* = 14 AD patients; *n* = 15 age-matched controls)	Pain ratings combined with functional MRI during noxious mechanical stimulation	Similar subjective pain ratings among groups; preserved activation of medial and lateral pain pathways (insula, anterior cingulate, somatosensory cortices); no reduction in pain-related brain activity in AD; in some regions, increased amplitude and duration of activation compared to controls	Pain processing is preserved at both sensory and affective neural levels in Alzheimer’s disease, challenging the assumption of reduced pain perception and highlighting risk of undertreatment
Combined psychophysical and resting-state fMRI study in Alzheimer’s disease [70]	*n* = 23 AD; *n* = 23 age-matched controls	Thermal perception (warmth, mild, moderate pain ratings) and RSFC across pain-related networks	AD associated with increased thermal thresholds for warmth and mild pain but preserved moderate pain unpleasantness; reduced RSFC between posterior insula and amygdala–somatosensory cortex; altered coupling between prefrontal and cingulate regions in controls only; disrupted correspondence between psychophysical responses and network connectivity	Altered integration between sensory, affective and descending modulatory pain networks in AD may underlie reduced pain reporting and contribute to underuse of analgesic treatment
Neurophysiological study in AD assessing cognitive status, EEG activity, sensory thresholds, and autonomic responses [71]	*n* = 30 non-consecutive communicative AD patients	Pain detection thresholds, pain thresholds, and heart rate responses to nociceptive stimulation and anticipation	No correlation between AD severity and sensory-discriminative measures (detection and pain thresholds); significant correlation between cognitive impairment severity and autonomic responses to pain (heart rate reactivity and anticipatory responses), associated with EEG slowing (delta/theta activity)	Strong evidence for dissociation between preserved sensory-discriminative pain processing and cognitively modulated autonomic–affective responses, suggesting progressive disruption of integrative pain networks in AD
Video-based observational study comparing self-report and coded nonverbal pain behaviors [72]	*n* = 58; 29 with cognitive impairment	Self-report pain ratings and observational coding of facial expressions, guarding behavior, and pain-related movements	Pain increased with physical activity across both groups; cognitively impaired participants showed heightened facial reactivity and more pronounced behavioral indicators (especially guarding); only modest correlations between self-report and behavioral measures, indicating partial dissociation between pain modalities	Self-report and behavioral pain measures capture complementary but non-overlapping aspects of pain in cognitively impaired elders, supporting multimodal assessment strategies for clinically relevant pain detection
Multimodal psychophysiological study using calibrated noxious electrical stimulation [73]	Dementia patients (*n* = 35) and age-matched healthy controls (*n* = 46)	Subjective ratings, NFR and SSR	Dementia patients showed preserved pain ratings when able to respond but reduced reporting capacity; increased facial pain responses; decreased NFR threshold indicating enhanced reflex sensitivity; and attenuated autonomic reactivity compared to controls	Dementia is associated with a dissociation across pain systems, with enhanced reflexive and expressive responses but reduced autonomic integration, supporting a multidimensional and non-uniform alteration of pain processing and underscoring the need for multimodal pain assessment
Quasi-experimental study [74]	Older adults with mild AD (*n* = 27) and healthy controls (*n* = 36)	Facial expression of pain (FACS) during innocuous and painful stimuli	Facial expressions varied appropriately with stimulus intensity; no significant differences between AD and control groups in facial pain reactivity	Reflexive facial pain expression is preserved in mild AD, supporting the validity of behavioral coding systems for pain assessment independent of cognitive status
Experimental psychophysiological study in Alzheimer’s disease patients [75]	20 non-consecutive communicative patients (mean age 68.6 years ± 3.8 SD) with AD and 20 healthy subjects (mean age 68.8 years ± 3.2 SD)	Pain perception ratings and autonomic responses during low- and high-intensity electrical stimulation	At threshold stimulation, AD patients showed normal pain perception but blunted autonomic responses; at supra-threshold stimulation, autonomic responses became comparable to controls while pain perception was reduced; overall increased thresholds for both autonomic activation and pain tolerance in AD	AD is associated with intensity-dependent dissociation between sensory and autonomic pain processing, suggesting altered gain control and elevated thresholds for both pain perception and physiological reactivity

PAIN-AD, Pain Assessment in Advanced Dementia; PACSLAC, Pain Assessment Checklist for Seniors with Limited Ability to Communicate; NPI, Neuropsychiatric Inventory; BPSD, behaviors and psychological symptoms of dementia; NPS, neuropsychiatric symptoms; EEG, electroencephalogram; FACS, Facial Action Coding System; NFR, nociceptive flexion reflex; SSR, sympathetic skin response.

**Table 2:** Representative preclinical evidence of dissociated pain processing and pain–cognition interactions in Alzheimer-like and chronic pain models.

Study	Design/Population	Pain Assessment	Key Findings	Clinical Significance
Chronic pain models: effects on cognition and affective behavior				
Preclinical mouse study evaluating long-term effects of acute single vs. repetitive inflammatory pain during infancy [27]	CD1male and female mice; pain model with single tail clip or repetitive needle pricks	Anxiety-like behavior (elevated plus maze) and spatial learning (Morris water maze)	Repetitive neonatal pain exposure increased anxiety-like behaviors in adolescence, reflected by reduced open-arm exploration and increased risk-assessment behaviors, while spatial learning remained unaffected; single acute-pain exposure produced no significant behavioral changes.	Early repetitive pain exposure induces persistent affective vulnerability without parallel cognitive impairment, indicating that pain-related developmental plasticity selectively alters emotional circuits.
Preclinical rat study on acute inflammatory pain and morphine on attentional performance [28]	Sprague Dawley rats; formalin-induced pain and 5-CSRTT with graded morphine administration	Attentional performance (task omissions) under pain and pharmacological modulation	Acute inflammatory pain significantly impaired attention (increased omissions); high-dose morphine (6 mg/kg) similarly impaired performance (sedation effect), whereas an analgesic dose (3 mg/kg) improved attentional performance without major sedation.	Pain disrupts attentional processing, and its cognitive impact can be partially reversed by appropriate analgesic dosing, highlighting the need to distinguish analgesic from sedative drug effects in pain management.
Preclinical mouse model of neuropathic pain stratified by behavioral traits (sociality, anxiety, depressive-like behavior) [80]	Swiss albino male mice; partial sciatic nerve ligation or sham surgery to induce neuropathic pain	Mechanical hypersensitivity, emotional-like behavior, cognitive-like performance, and central amygdala activity	Spontaneous central amygdala activity correlated with sociability traits; low-sociable/high-anxious/low-depressive phenotypes showed increased nociceptive hypersensitivity, whereas high-sociable/anxious/depressive-like phenotypes exhibited stronger emotional and cognitive impairments; nociceptive, emotional, and cognitive manifestations were dissociable.	Neuropathic pain phenotypes are determined by pre-existing behavioral and molecular signatures, supporting a dissociation between sensory hypersensitivity and affective–cognitive alterations driven by amygdala-related neuroimmune and transcriptional mechanisms.
Preclinical mouse model of neuropathic pain with cognitive and motivational behavior assessment [81]	Swiss albino male mice; partial sciatic nerve ligation–neuropathic pain	Mechanical allodynia, thermal hyperalgesia, anxiety- and depressive-like behavior, anhedonia, object-recognition memory, and operant motivation	Neuropathic pain associated with increased anxiety- and depressive-like behaviors, anhedonia, memory impairment, and reduced motivation; pregabalin improved nociceptive, anxiety-like, anhedonic and memory deficits, but did not reverse depressive-like behavior or motivational impairments.	Emotional and cognitive dimensions of chronic pain are only partially responsive to analgesic treatment, supporting a dissociation between nociceptive relief and affective-motivational recovery and emphasizing the need for multidimensional outcome measures in pain research.
Longitudinal experimental study evaluating sustained attention before and after chronic pain induction [82]	Lister Hooded rats; CFA-induced monoarthritis	Mechanical sensitivity (von Frey) and 5-CSRTT	Chronic pain-induced persistent attentional deficits (increased errors and omissions); analgesic treatment (carprofen) reduced nociceptive sensitivity, but did not improve attentional performance.	Attentional impairment in chronic pain is not solely driven by ongoing nociceptive input, but reflects sustained alterations in neural systems underlying attention, highlighting dissociation between pain relief and cognitive recovery.
Preclinical rat study across lifespan with neuropathic pain [83]	3-, 10-, and 22-month-old rats; SNI model	Open-field, elevated-plus-maze, forced swim test, working-memory maze, Morris water maze, spatial reversal	Neuropathic pain effects were age-dependent: increased anxiety across age groups (enhanced in young and old), depressive-like behavior and cognitive. impairment primarily in middle-aged animals; aging alone impaired cognitive performance, while pain selectively exacerbated deficits, depending on domain and age.	The impact of chronic pain on affective and cognitive domains is dynamically modulated by age, with domain-specific vulnerability, highlighting the fact that pain-related behavioral alterations depend on both neurobiological aging and network-specific susceptibility.
Translational study combining preclinical mouse model with human neuroimaging in chronic pain patients [84]	C57BL/6 male mice; SNI model; patients with CBP, CRPS, OA	Behavioral assays (fear extinction, anxiety), molecular markers (ERK signaling, neurogenesis), synaptic plasticity, and hippocampal volume in humans	Neuropathic pain impaired fear extinction and increased anxiety in mice, associated with reduced ERK signaling, decreased neurogenesis, and altered synaptic plasticity in the hippocampus; human patients with chronic pain (CBP, CRPS) showed reduced hippocampal volume, unlike OA patients.	Chronic pain is associated with structural and functional hippocampal alterations that link impaired cognitive–emotional processing to underlying neuroplastic changes, supporting a neurobiological substrate for pain-related affective and memory dysfunction.
Preclinical rodent study [85]	Sprague Dawley male rats and male C57 mice; SNI model	Behavioral memory tasks (working memory, short-term memory), hippocampal LTP, frequency facilitation, synaptic bouton density, TNF-α levels	SNI-induced working and short-term memory deficits associated with impaired CA3–CA1 synaptic plasticity, reduced presynaptic bouton density, and elevated TNF-α in hippocampus, CSF and plasma; TNF-α administration replicated, while inhibition or TNF receptor 1 deletion prevented both cognitive and synaptic deficits.	Neuroinflammatory signaling via TNF-α represents a key mechanistic link between peripheral neuropathic pain and hippocampal-dependent cognitive impairment, suggesting shared inflammatory pathways underlying pain and memory dysfunction.
Preclinical rat study using SNI model with chronic electrophysiological recordings [86]	Sprague Dawley rats; SNI model	Figure-eight spatial alternation task, non-sample-to-match T maze	SNI-induced chronic pain impaired working-memory performance, altered mPFC population firing during decision points, increased phase-locking to hippocampal theta rhythm and reduced information transfer within the fronto-hippocampal circuit, with oscillatory patterns predicting correct vs. incorrect trials.	Chronic pain disrupts large-scale fronto-hippocampal network dynamics underlying working memory, indicating that cognitive deficits arise from impaired inter-regional communication and altered neural synchrony, rather than isolated regional dysfunction.
Preclinical rat study [87]	Sprague Dawley rats; SNI model	Mechanical and thermal hypersensitivity (von Frey, acetone tests), progressive ratio responding, and 5-CSRTT performance	SNI induced persistent sensory hypersensitivity without affecting motivation for food reward; however, a delayed and progressive attentional deficit emerged (reduced accuracy, slower responses, increased omissions) during 5-CSRTT performance.	Chronic neuropathic pain selectively impairs attentional processing while sparing motivational drive, indicating domain-specific cognitive vulnerability and time-dependent emergence of pain-related executive dysfunction.
Preclinical rat study with chronic pain using pharmacological interventions (amitriptyline vs. lornoxicam) [88]	Sprague Dawley rats; L5 spinal nerve transection	Mechanical allodynia, depressive-like behavior, spatial-learning and memory tasks	Neuropathic pain induced mechanical allodynia, depressive-like behavior, cognitive impairment and reduced hippocampal BDNF expression; amitriptyline reversed both affective and cognitive deficits while restoring BDNF levels, whereas lornoxicam reduced pain without improving cognition or BDNF expression.	Cognitive impairment in neuropathic pain is linked to hippocampal neurotrophic dysregulation, and recovery of cognitive function depends on modulation of neuroplasticity, rather than analgesia alone.
A dual in vivo model of chronic neuropathic pain and cognitive impairment [43]	Naturally aged male Sprague Dawley rats combining scopolamine-induced transient cognitive impairment (AD-like features) and unilateral sciatic nerve ligation–induced neuropathic pain	Thermal nociceptive tests and mechanical sensitivity assays; behavioral and clinical monitoring. Pharmacological modulation with an EU-GMP certified *Cannabis sativa* L. strain	The therapy produced robust time-dependent analgesia in thermal nociceptive tests, with enhanced effects when combined with donepezil and tramadol. Mechanical sensitivity was minimally affected.	Suggests that modulation of the endocannabinoid system may exert both analgesic and neuroprotective effects in conditions combining cognitive impairment and chronic neuropathic pain. Supports the relevance of multi-targeted approaches for dementia with comorbid pain, although findings are based on an induced, non-transgenic AD-like model.
AD models combined with experimentally induced chronic pain				
Preclinical study investigating whether dendritic spine dysgenesis and instability in the ACC contribute to reduced pain sensitivity in inflammatory pain conditions in an AD model [89]	5 × FAD Alzheimer’s disease mice subjected to CFA-induced inflammatory pain	Mechanical pain measurement (von Frey test); CatWalk gait analysis; hot plate test	5 × FAD mice showed attenuated mechanical allodynia associated with reduced excitatory neuronal activity, delayed ACC responses and dendritic spine loss in ACC pyramidal neurons.	Suggests that AD-related synaptic dysfunction within ACC circuits may impair affective-motivational pain processing and contribute to altered pain expression in AD. Supports a mechanistic link between dendritic spine pathology and diminished pain responsiveness in AD models.
Preclinical AD mice study with chronic monoarthritis [29]	APP/PS1 mice with chronic monoarthritis induced by intra-articular CFA injections	Behavioral pain responses and cognitive testing (learning and memory models)	Chronic monoarthritis pain accelerated cognitive impairment selectively in APP/PS1 mice and was associated with increased hippocampal NR2B expression and altered NR2B/NR2A ratio.	Supports bidirectional interactions between chronic pain and AD-related neurodegeneration, suggesting that pain may exacerbate cognitive decline through higher-order neurobiological mechanisms.
Preclinical AD mice study with inflammatory pain using pharmacological intervention (naloxone) [90]	4- to 7-month-old adult male and female double-mutant APPswe × PS1.M146V (TASTPM) transgenic mice, Carrageenan model of inflammatory pain	Acute noxious thermal stimulation; nociceptive threshold testing; pharmacological modulation with naloxone	Age-dependent thermal hypoalgesia associated with increased spinal inhibitory tone and central/spinal amyloid pathology, reversed by naloxone, implicating endogenous opioid mechanisms.	Provides direct evidence that AD-related pathology can alter nociceptive processing at spinal and central levels, supporting the hypothesis that reduced pain responses in AD may reflect neurobiological changes in pain pathways, rather than impaired reporting alone.
APP/PS1 chronic pain interaction study [91]	APP/PS1 mice subjected to partial sciatic nerve ligation (neuropathic pain) or CFA-induced inflammatory pain	Mechanical allodynia (von Frey filaments); behavioral assessment of cognition (NOR, Morris water maze, Y-maze, passive avoidance) and affective-like behavior	Both neuropathic and inflammatory pain reduced pain thresholds and significantly worsened learning, memory, and depression-like behaviors in APP/PS1 mice. Chronic pain was associated with increased hippocampal and cortical neuroinflammation and enhanced AD pathology.	Demonstrates bidirectional interaction between chronic pain and AD pathology, suggesting that persistent pain exacerbates neuroinflammation and accelerates cognitive and molecular AD-related deficits
3xTg-AD thermal nociception study [92]	Sex- and age-stratified 3xTg-AD transgenic mice (2–15 months) compared with non-transgenic controls	Plantar test assessing thermal withdrawal reflexes; evaluation of sensory-discriminative and affective/emotional responses	Sensory-discriminative thermal withdrawal thresholds were largely preserved across disease stages compared to controls. However, sex-specific differences were observed: females showed increased sensitivity at premorbid stages, while males exhibited enhanced emotional reactivity to thermal nociception.	Demonstrates that AD-related pathology does not uniformly alter nociceptive thresholds, but affects sex- and stage-dependent components of pain processing, highlighting dissociation between sensory and affective dimensions of pain in AD models.

5-CSRTT, 5-choice serial reaction time task; SNI model, spared nerve injury; CBP, chronic back pain; CRPS, complex regional pain syndrome; OA, knee osteoarthritis; ERK signaling, extracellular signal-regulated kinase signaling; LTP, long-term potentiation; TNF-α, tumor necrosis factor-α; CA3–CA1, subregion of the hippocampus; CSF, cerebrospinal fluid; mPFC, medial prefrontal cortex; BDNF, brain-derived neurotrophic factor; ACC, anterior cingulate cortex; CFA, Complete Freund adjuvant; NOR, Novel Object Recognition Test; NR2A and NR2B, NMDAR subunits; NMDAR, N-methyl-D-aspartic acid receptor.

## Data Availability

No new data were created or analyzed in this study. Data sharing is not applicable to this article.

## Abbreviations

The following abbreviations are used in this manuscript:

AD	Alzheimer’s disease
Aβ	beta-amyloid
IASP	International Association for the Study of Pain
PAIN-AD	Pain Assessment in Advanced Dementia
PACSLAC	Pain Assessment Checklist for Seniors with Limited Ability to Communicate
NPI	Neuropsychiatric Inventory
BPSD	behaviors and psychological symptoms of dementia
NPS	neuropsychiatric symptoms
EEG	electroencephalogram
FACS	Facial Action Coding System
NFR	nociceptive flexion reflex
SSR	sympathetic skin response
5-CSRTT	5-choice serial reaction time task
SNI model	spared nerve injury
CBP	chronic back pain
CRPS	complex regional pain syndrome
OA	knee osteoarthritis
ERK signaling	extracellular signal-regulated kinase signaling
LTP	long-term potentiation
TNF-α	tumor necrosis factor-α
CA3–CA1	subregion of the hippocampus
CSF	cerebrospinal fluid
mPFC	medial prefrontal cortex
BDNF	brain-derived neurotrophic factor
APS	Abbey Pain Scale
iNOS	inducible Nitric Oxide Synthase
IL-1β	Interleukin-1 beta
IL-6	Interleukin-6
IL-12	Interleukin-12
IL-23	Interleukin-23
